# Identifying research priorities for infection prevention and control. A mixed methods study with a convergent design

**DOI:** 10.1177/17571774241230676

**Published:** 2024-02-20

**Authors:** MP Smiddy, E Burton, L Kingston, T Thomas Poovelikunnel, M Moyo, A Flores

**Affiliations:** 1 School of Public Health, 8795 University College Cork, Cork, Ireland; 2 Pharmacy Department, Bon Secours Hospital, Cork, Ireland; 3 Department of Nursing and Midwifery, 8808 University of Limerick, Limerick, Ireland; 4 11388 Office of the National Director Health Protection, HSE - Health Protection Surveillance Centre, Dublin, Ireland; 5 Faculty of Nursing and Midwifery, RCSI, Dublin; 6 7425 Department of Social Sciences and Nursing, Solent University, Southampton, UK; 7 6831 Infection Prevention and Control Department, Kings College Hospitals Foundation NHS Trust, London, UK

**Keywords:** Infection prevention, infection control, research, research priorities, quality improvement, mixed methods research

## Abstract

**Background:**

Meaningful research creates evidence for Infection Prevention and Control (IPC) practice.

**Aim:**

To establish Infection Prevention Society (IPS) members’ research priorities to support future research projects.

**Methods:**

A mixed methods convergent parallel design incorporating a cross-sectional survey of IPS members (2022–2023), and focus group findings from the IPS Consultative Committee, (October 2022). Quantitative data were analysed using descriptive statistics. Qualitative data were transcribed verbatim, entered into NVivo 12, and analysed using a thematic analysis approach.

**Findings/Results:**

132 IPS members responded to the survey, including 120 (90.9%) nurses. The three most prevalent priorities were: Quality Improvement and Patient Safety (*n* = 84, 16.1%); IPC Training and Education (*n* = 77, 14.8%); and IPC Evidence-based Guidelines (*n* = 76, 14.6%). Analysis of the focus group transcripts identified six emergent themes ‘Patient Centred Care’, ‘Training and Education’, ‘IPC Role and Identity’, ‘IPC Leadership’, ‘IPC is Everyone’s Responsibility’, and ‘Research Activity’. Triangulation of findings demonstrated concordance between quantitative and qualitative findings with Quality Improvement and Patient Safety (QIPS) and Training and Education identified as priority research areas.

**Discussion:**

This study highlights the necessity of developing support systems and incorporating research priorities in QIPS, as well as Training and Education. The findings of this study align with the recommended core competencies and components for effective infection prevention and control programs, making them relevant to QIPS initiatives. The outcomes of the study will serve as a valuable resource to guide the IPS Research and Development Committee in delivering practical support to IPS members.

## Background

Meaningful high-quality research creates the evidence base for Infection Prevention and Control (IPC) practice. The importance and value of IPC principles in pandemic planning and the delivery of safe patient care have been highlighted since the inception of the SARS-CoV-2 pandemic in 2020 (Loveday and Wilson, 2021). Loveday and colleagues (2009) previously conducted Infection Prevention Society (IPS) research in this area indicating that human behaviour, infection prevention interventions, and health policy were the priorities at the time. In view of the developments of recent years, it was timely to establish the current research priorities of IPS members to inform the research of the Society and the prioritisation of research funding to support IPS practitioner-led research in relevant areas.

Lacotte and colleagues (2020) indicated three top research priorities for IPC as, the identification of barriers and facilitators to implementing effective IPC programmes, the impact of overcrowding on the transmission of healthcare-associated infection (HCAI), and the evaluation of the healthcare environment related to the reduction of HCAI and the emergence of antimicrobial resistance. This is consistent with the World Health Organization (WHO) priorities, which relate to expertise, resources, education, IPC as a performance indicator, visibility, advocacy, and support (Allengranzi et al., 2017). In addition, early accurate diagnosis, education of healthcare workers, patients and carers, and the evaluation of antimicrobial stewardship (AMS) interventions were highlighted as priority areas for IPC research (Wilson et al., 2019). However, in the intervening years much has changed in the application and practice of IPC. Thus, the aim of this research is to establish IPS members’ current research priorities.

## Methods

### Study design

This research used a pragmatic mixed methods parallel convergent design (Cresswell and Plano Clarke, 2011). A cross-sectional survey of IPS member responses (February–March 2023), and focus group findings from a meeting with the IPS Consultative Committee (October 2022) are included. A mixed methods approach was taken, triangulating both quantitative and qualitative findings to gain a more complete understanding of IPS member research priorities.

### Data collection

#### Quantitative

A survey collecting IPS member research priorities was conducted from February to March 2023. The survey was adapted from previous IPC research priority studies and focused on the following eight areas: IPC Interventions and Guidelines, IPC Training and Education (T&E), Healthcare Infection (HCI) Surveillance and Monitoring, HCAI and AMS, Behavioural Science, Sustainability, and Quality Improvement and Patient Safety (QIPS). The survey was sent to all IPS members (*N* = 2415). Data were collected using Qualtrics software (2005).

#### Qualitative

The focus group was conducted with members of the Management Executive Group, including Branch Coordinators (*N* = 16), and members of the IPS Special Interest Groups (*N* = 24). The discussion was initiated with a participant information presentation based on the eight possible research priorities used in the quantitative survey, and a semi-structured topic guide. The focus group was recorded and transcribed verbatim. Transcripts were imported into NVivo 12 (QSR International, 2017).

### Analysis

#### Quantitative

Descriptive statistics were used to summarise survey question responses. Group comparisons were performed using the Chi-square test except for where priorities were rarely indicated in which case Fisher’s exact test was used.

#### Qualitative

Analysis of the focus group transcripts was implemented using a thematic analysis approach, (Braun and Clarke, 2006). NVivo 12 qualitative data management software was used to support data storage, organisation, and analysis (QSR International, 2017). The transcript was coded by one of the focus group facilitators [MS] and separately by a second researcher [EB]. Differences were reviewed by a second focus group facilitator [AF] and another member of the research team [LK] as a method of verification and validation of analysis. Agreements on coding were reached through discussion and consensus. Researcher reflexivity was considered and explored before and during the research process, (Supplemental File (SF) 1).

### Triangulation

Triangulation of the results using a convergent design (Cresswell and Plano Clarke, 2011) was implemented, (SF 2).

### Ethical approval

Ethical approval was granted for this research from the University of West London, College of Nursing, Midwifery & Health Ethics Committee, Ref No. 1387 and consent was obtained from the participants.

### Process evaluation

The study design was mapped to the framework guiding evidence-informed priority-setting processes for health research (Tan et al., 2022), and guided by the reporting guidelines for priority setting of health research (REPRISE), (Tong et al., 2019) (SF 3) and the COnsolidated criteria for REporting Qualitative (COREQ) research checklist (Tong et al., 2007) (SF 4).

## Results

### Quantitative society-wide survey

Responses to the survey were received by 132 of 2415 (correct as of October 2022) IPS members, giving a 5% (95% CI: 1.4–8.6) response rate. Participant characteristics are presented in Table 1.

**Table 1.** Characteristics of survey respondents.

Factor	Responses (*n*)	Category	Number (%)
Profession	132	Nurse	120 (91)
		Other	12 (9)
Work sector	131	Primary only	21 (16)
		Secondary only	35 (27)
		Academia only	8 (6)
		Other ^(+)^	67 (51)
Work location	130	England	107 (82)
		Wales	11 (8)
		Scotland	4 (3)
		Republic of Ireland	4 (3)
		Northern Ireland	1 (1)
		Other	3 (2)
Years’ experience	130	<5 years	25 (19)
		5–10 years	30 (23)
		>10 years	75 (58)
Senior management role	127	Yes	75 (59)
		No	52 (41)

(+) Including respondents working in more than one sector.

The remaining survey questions related to the research priorities of the respondent, their organisation, and respondents’ views on research priorities for IPS, Table 2.

**Table 2.** Member research priorities and comparison of priorities by IPC management position.

Member research priorities				Comparison of priorities by IPC management position		
Question	Responses (*n*)	Category	Number (%)	Not management	Management	*p*-value
Areas you prioritise ^(*)^	132	Quality improvement/safety	84 (64)	31/52 (60%)	53/75 (71%)	0.20
		IPC training & education	77 (58)	36/52 (69%)	37/75 (49%)	0.03
		Behavioural science	70 (53)	29/52 (56%)	38/75 (51%)	0.57
		IPC interventions & guidelines	68 (52)	26/52 (50%)	39/75 (52%)	0.83
		Sustainability	56 (42)	26/52 (50%)	28/75 (37%)	0.16
		HCAI/AMS	48 (36)	17/52 (33%)	29/75 (39%)	0.49
		HCI surveillance & monitoring	36 (27)	13/52 (25%)	22/75 (29%)	0.59
		Other	7 (5)	2/52 (4%)	4/75 (5%)	0.70
Your organisations priorities ^(*)^	77	Other	33 (43)	14/28 (50%)	18/47 (38%)	0.32
		HCAI/AMS	22 (29)	9/28 (32%)	12/47 (26%)	0.54
		HCI surveillance & monitoring	19 (25)	7/28 (25%)	11/47 (23%)	0.88
		IPC interventions & guidelines	17 (22)	7/28 (25%)	10/47 (21%)	0.71
		IPC training & education	12 (15)	4/28 (14%)	8/47 (17%)	0.76
		Quality improvement/safety	13 (17)	3/28 (11%)	10/47 (21%)	0.24
		Sustainability	11 (14)	4/28 (14%)	7/47 (15%)	0.94
		Behavioural science	8 (10)	1/28 (4%)	6/47 (13%)	0.19
IPS research priorities ^(*)^	113	IPC interventions & guidelines	44 (39)	20/45 (44%)	23/63 (37%)	0.41
		Other	43 (38)	15/45 (33%)	25/63 (40%)	0.50
		IPC training & education	33 (29)	19/45 (42%)	13/63 (21%)	0.02
		Behavioural science	28 (25)	14/45 (31%)	13/63 (21%)	0.22
		Sustainability	28 (25)	16/45 (36%)	10/63 (16%)	0.02
		Quality improvement/safety	25 (22)	11/45 (24%)	14/63 (22%)	0.79
		HCAI/AMS	23 (20)	7/45 (16%)	14/63 (22%)	0.39
		HCI surveillance & monitoring	11 (10)	5/45 (11%)	6/63 (10%)	0.79

(*) Participants could respond in more than one category. Percentage values will not add to 100%.

The comparisons between work sectors and level of experience resulted in no statistically significant differences between organisational priorities or research interests. There were significant differences between those in non-senior positions and senior management roles relating to T&E with those in senior management less likely to indicate T&E as a priority area. This is consistent with non-management participants significantly more supportive of T&E as research than those in management, Table 2. There was no significant difference between participants’ perceived organisational research priorities, Table 2.

There was a significant difference between non-senior and senior management participants in relation to sustainability as a research priority with senior management staff less likely to indicate sustainably as a priority area, Table 2. Both groups indicated sustainability as one of the less prominent organisational priorities, Table 2.

### Qualitative focus group findings

21 members from the IPS Management Executive Group and Consultative Committee Group participated in the focus group. The majority were nurses (*n* = 19) with most having over 10 years’ experience (*n* = 16) with 15 indicating they held a senior IPC role.

## Themes

### Patient centred care

Patient centred care was a recurring theme, (P1, P6, P11, P12). Placing the patient at the centre of IPC in practice was viewed as important, *‘putting the patient at the heart of everything that we do’,* (P1). The COVID-19 pandemic was perceived to have had an adverse effect on patient focussed care and participants indicated the importance of refocussing on the patient, (SF 5).

### Training and education

#### Healthcare workers

The importance and value of training and education were highlighted as a priority for IPC (P1, P2, P11, P12). Concern was evident related to the impact of the COVID-19 pandemic particularly the pressure to respond and adapt, changing guidelines and loss of autonomy, *‘a lot of the basics of IPC have been lost’* (P2). Informal training and education were highlighted as important to supporting and developing clinical staff, (SF 5).

#### Infection prevention and control practitioners

Issues such as professional development for IPCNs, attracting new entrants and retaining staff were viewed as key issues, *‘how do we keep them?’* (P11). Formal professional development for IPCNs was indicated as important and the content of academic programmes needing to be fit for purpose, (SF 5).

### Infection prevention and control role and identity

Participants identified a lack of defined role and identity as challenging in the application of IPC. The COVID-19 pandemic was perceived to influence the interpretation and understanding of an ideal IPC service. Diversity of job titles, roles, and responsibilities within the IPS was perceived as challenging in terms of wider identity associated with IPC, *‘we all see each other, somebody might have said they’re an Infection Control Nurse, somebody else said an Infection Prevention Nurse’,* (P5), (SF 5).

The challenges of IPC application, associated guidance, and clinical adaptations during the COVID-19 pandemic were perceived to negatively impact practitioner’s ability to risk assess and apply clinical judgement, impacting autonomy and identity, (SF 5).

### Infection prevention and control leadership

IPC was viewed by participants as a multidisciplinary leadership role (P1, P2, P5, P6, P12). The concept of development of and integration of IPC leadership was explored further in considering inclusion of IPC in integrated care systems, (SF 5).

### Infection prevention and control is everyone’s responsibility

A lack of autonomy and accountability from non-IPC healthcare workers in relation to application of IPC was discussed, (P9, P11, P13), ‘*they don’t want to take it back onto themselves’,* (P13). Integration of IPC into routine patient care was viewed as a priority and fundamental to safe patient care, ‘*not IPC being seen as a separate job’,* (P9), (SF 5).

### Infection prevention and control research activity

Implementation of research as part of the IPS role was viewed positively but not without challenges (P1, P2, P5, P12, P20). Engagement, successful completion, support, and application of research findings were viewed as enablers and encouraged participants to pursue research activities. Supportive organisational culture encouraged participants to engage with research however, this was not everybody’s experience, *‘I think a lot depends on where you’ve worked’,* (P1). Time, confidence, and lack of support were identified as barriers to implementation, *‘time is our biggest challenge’,* (P5).

Concern regarding training of IPC professionals and the dissemination of research outcomes was evident, *‘how many people have had training in research?’* (P5). Meaningful engagement of patients in research was highlighted as a challenge and identifying patients research priorities was viewed as important, *‘how can we involve more patients in deciding what all the priorities are’,* (P2), (SF 5).

### Triangulation of quantitative and qualitative data analysis

**Figure 1.**
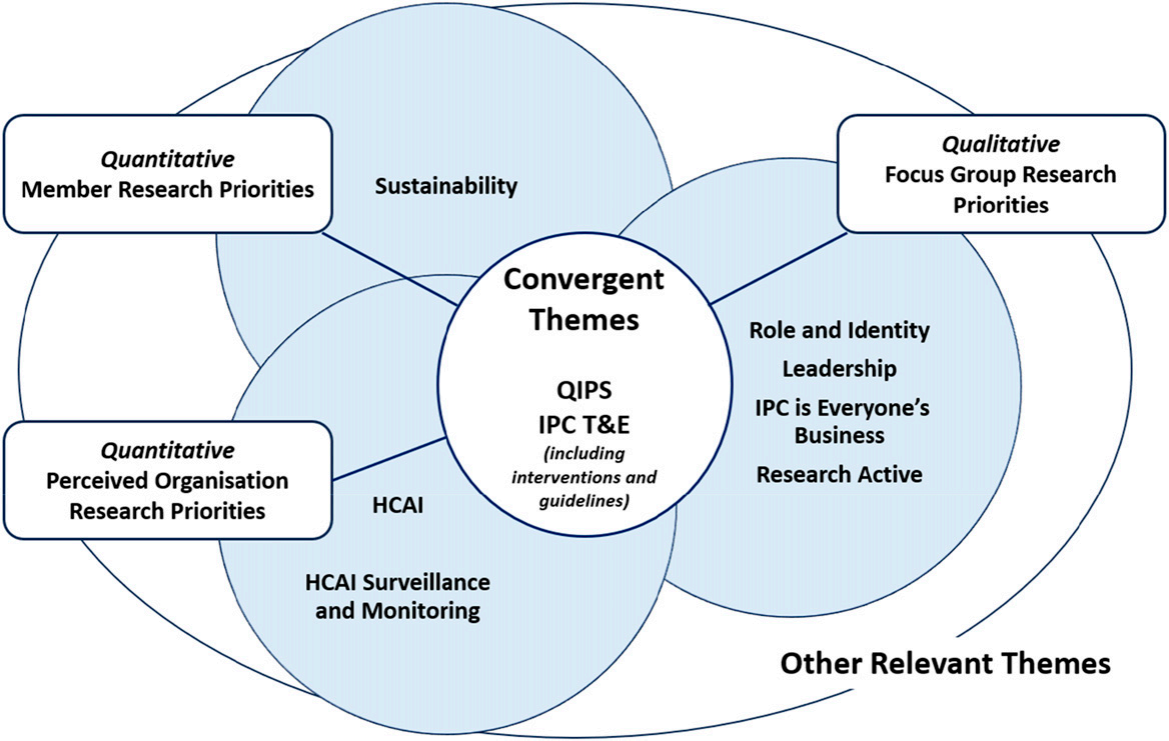
Triangulated findings from member survey and focus group.

Triangulation of results is presented in Figure 1, (SF 5). QIPS and IPC T&E were common to both studies. ‘Patient Centred Care’ is embedded into the QIPS category as the focus was on the patient and their safety. Sustainability was identified as an important area of research for IPS members and participants perceived that HCAI/AMS and HCAI Surveillance and Monitoring were of priority to their organisations. ‘Role and Identity’, ‘Leadership’, ‘IPC is Everyone’s Business’, and the ability to be ‘Research Active’ were additional themes from the focus group. The participants indicated IPC being everyone’s responsibility needs to be strengthened and implemented. While research activity as part of the IPC role was positively perceived, confidence and time to engage emerged as barriers to implementation.

## Discussion

The aim of this research was to identify members’ IPC research priorities to inform the work of the IPS. Consistent with Curran et al. (2021), we found that IPS members were supportive of QIPS as a research priority for the society, but it was not perceived to be the most common research priority of the IPS or the participants’ organisations. QIPS was framed differently by the focus group participants who spoke about putting the patient at the centre of care delivered. Quality improvement is a core competency for the implementation of IPC activities (IPS EPCD, 2021; WHO, 2020). This focus on quality improvement and patient safety is supported (Allegranzi et al., 2017; Hessels et al., 2023). The breath of QIPS in IPC can be viewed to incorporate all the findings of this research even though they did not directly converge.

IPC T&E in association with interventions and guidelines was identified as a research priority in both studies (Robinson et al., 2023). Interventions and guidelines featured in the focus group discussion and are included consistently under T&E. T&E was identified as important to all healthcare workers and a lack of appropriate education and training has been indicated as detrimental to implementation of effective IPC (Burnett et al., 2023). Specific emphasis emerged during the qualitative research which focussed on the training needs of IPC professionals. Tsioutis and colleagues (Tsioutis et al., 2020) identified broad heterogeneity in training programmes across Europe and recommend establishment of IPC T&E programmes with agreed commonalities. More senior participants in the quantitative survey prioritised training and education significantly less than their more junior colleagues. Increased seniority, cognitive flexibility, and professional experience were positively associated with the ability to cope with stressors during the pandemic (Kruczek et al., 2020), which may have influenced the perceptions of more senior staff. Professional experience is associated with increased resilience in healthcare workers (Abdul Salem et al., 2023).

Sustainability emerged as a priority for the participants of the survey. Sustainability is essential to the provision of quality healthcare (Putnis and Neilson, 2022). IPC considerations to improve sustainability, include the built environment, appropriate use of resources, education, and implementation strategies. Commensurate with the convergent result of QIPS, HCAI/AMS and HCI surveillance and monitoring were perceived as priorities, consistent with WHO recommendations (Storr et al., 2017).

Participants in the focus group indicated that ‘Role and Identity’, ‘Leadership’, ‘IPC is Everybody’s Business’, and the importance of being ‘Research Active’ were important research priorities. The theme ‘Role and Identity’ questions the ideal structure of IPC services in an environment with broad role diversity challenged by varied levels of autonomy to support practice. Appropriate resources, consistency, clarity, and ease of application in evidence-based guidelines, as well as appropriate T&E and organisational support are recommended to address the challenges associated with the IPC role and identity (Robinson et al., 2023).

‘Leadership’ was indicated as important to focus group participants consistent with the IPS core competencies (Infection Prevention Society Education Practice and Development Committee [EPDC], 2021) and perceived as valuable to support engagement at a multidisciplinary level. Leadership is essential for effective IPC and can occur at all levels of seniority (Gould et al., 2016a). This links effectively with the theme of ‘IPC is Everybody’s Business’ where participants viewed IPC being integrated into routine daily care across all disciplines. Actively embedding a sense of IPC ownership in all healthcare workers has been shown to improve service provision and reduce acquisition of HCAI (Gould et al., 2016b). Being ‘Research Active’ was viewed as important but challenging. Conflicting perceptions of research amongst IPC practitioners has been reported; however, quality improvement was indicated to be a core part of the IPC practitioner role (Burnett et al., 2023).

The mixed methods convergent design provides a deeper understanding of the question being explored. The use of a framework for the evidence-informed priority-setting processes for health research Tan et al. (2022), the REPRISE guideline (Tong et al., 2019), and the COREQ checklist (Tong et al., 2007) support the validity of the methodological approach. The methodology is embedded in the pre-existing evidence to maximise useability and application of findings. All IPS members were invited to take part in the survey; however, only a 5% response rate was achieved. There were some discrepancies as well as similarities in the triangulated results; however, all results can be mapped to the main finding of QIPS. The single focus group with senior IPS members is a limitation and additional focus groups with more representation from IPC practitioners at different levels of experience may have provided additional perspectives.

The findings of this research are consistent with the recommended core competencies and components for effective infection prevention and control programmes (IPS EPDC, 2021; Storr et al., 2017). QIPS was the consistent overarching result across both studies. The findings of this research will inform the focus of the IPS Research and Development Committee to support members with future research activities.

## Supplemental Material

Supplemental Material - Identifying research priorities for infection prevention and control. A mixed methods study with a convergent design Supplemental Material for Identifying research priorities for infection prevention and control. A mixed methods study with a convergent design by MP Smiddy, E Burton, L Kingston, TT Poovelikunnel, M Moyo and A Flores in Journal of Infection Prevention.

Supplemental Material - Identifying research priorities for infection prevention and control. A mixed methods study with a convergent design Supplemental Material for Identifying research priorities for infection prevention and control. A mixed methods study with a convergent design by MP Smiddy, E Burton, L Kingston, TT Poovelikunnel, M Moyo and A Flores in Journal of Infection Prevention.

Supplemental Material - Identifying research priorities for infection prevention and control. A mixed methods study with a convergent design Supplemental Material for Identifying research priorities for infection prevention and control. A mixed methods study with a convergent design by MP Smiddy, E Burton, L Kingston, TT Poovelikunnel, M Moyo and A Flores in Journal of Infection Prevention.

Supplemental Material - Identifying research priorities for infection prevention and control. A mixed methods study with a convergent design Supplemental Material for Identifying research priorities for infection prevention and control. A mixed methods study with a convergent design by MP Smiddy, E Burton, L Kingston, TT Poovelikunnel, M Moyo and A Flores in Journal of Infection Prevention.

Supplemental Material - Identifying research priorities for infection prevention and control. A mixed methods study with a convergent design Supplemental Material for Identifying research priorities for infection prevention and control. A mixed methods study with a convergent design by MP Smiddy, E Burton, L Kingston, TT Poovelikunnel, M Moyo and A Flores in Journal of Infection Prevention.
