# Supplementary material for: Identifying research priorities for infection prevention and control. A mixed methods study with a convergent design
Source: J Infect Prev. 2024 Feb 20;25(3):59–65. doi: 10.1177/17571774241230676 (PMC10998549; doi:10.1177/17571774241230676)
Supplement: Supplemental Material - Identifying research priorities for infection prevention and control. A mixed methods study with a convergent design [file sj-pdf-1-bji-10.1177_17571774241230676.pdf]

Supplemental File 1. Reflexivity Statements of the Qualitative Researchers

MS, (PhD, MPH, RM, RGN) is a university lecturer and researcher with a clinical background in infection prevention and control. She was the Communications Officer of the Infection Prevention Society Research and Development Group during the design, data collection and analyses phases pf this research. The researchers experience supports a deep understanding of the clinical challenges and the methodological rigour required in the research process which supports a nonbiased approach to the study.

EB, (BPharm, MPharm, MPSI) is a PhD scholar and clinical hospital pharmacist. She was an ordinary committee member of the Infection Prevention Society Research and Development Group during the design, data collection and analysis phases of this research. The researcher was not present at the focus group. The researcher's clinical and research experience in infectious disease therapeutics and qualitative methodology facilitates an impartial approach to this topic.

LK, (PhD, MSc, PG Dip. BSc, RGN, RM, RNT) is an Associate Professor and researcher at University of Limerick, Ireland. She was the Grants Officer of the Infection Prevention Society Research and Development Committee during the design, data collection and analyses phases of this research. The researcher's clinical and research experience in qualitative methodology facilitates an impartial approach and a deep understanding of the methodological rigour required in the research process which supports a nonbiased approach to the study.

AF (MSc, BSc Hons, RN) is the Director of Infection Prevention & Control at Kings College NHS Foundation Trust. She is on the Advisory Committee on Antimicrobial Prescribing, Resistance and Healthcare-associated Infection (APRHAI) and was the R&D Committee Co-ordinator of the Infection Prevention Society during the design, data collection and analyses phases of this research. She has over 20 years' experience in infection prevention and control research and clinical practice, which enables an understanding of this area of research.
