# Supplementary figures and images for: Identifying research priorities for infection prevention and control. A mixed methods study with a convergent design
Source: J Infect Prev. 2024 Feb 20;25(3):59–65. doi: 10.1177/17571774241230676 (PMC10998549; doi:10.1177/17571774241230676)

**Supplemental File 2. Mixed Methods Convergent Analysis, (Cresswell and Plano Clarke, 2011).**

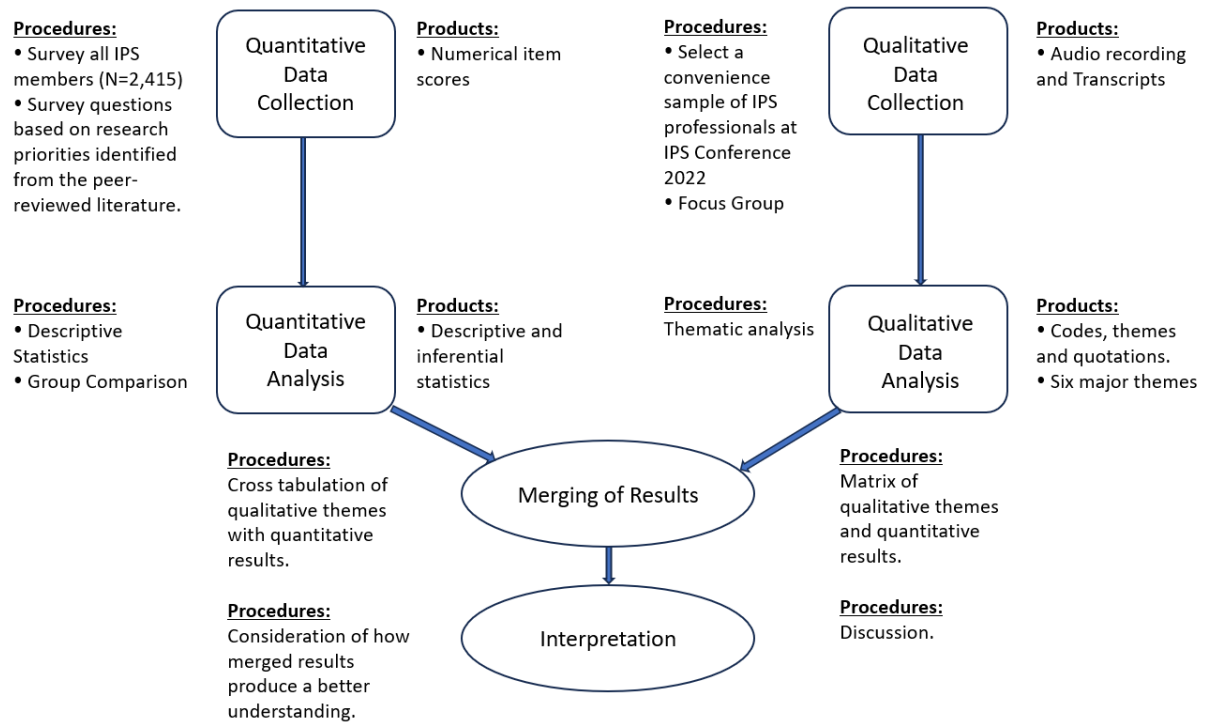

Supplement: Supplemental Material - Identifying research priorities for infection prevention and control. A mixed methods study with a convergent design [file sj-pdf-2-bji-10.1177_17571774241230676.pdf]
