# Supplementary material for: Identifying research priorities for infection prevention and control. A mixed methods study with a convergent design
Source: J Infect Prev. 2024 Feb 20;25(3):59–65. doi: 10.1177/17571774241230676 (PMC10998549; doi:10.1177/17571774241230676)
Supplement: Supplemental Material - Identifying research priorities for infection prevention and control. A mixed methods study with a convergent design [file sj-pdf-3-bji-10.1177_17571774241230676.pdf]

Supplementary File 3. Preliminary REPRISE guideline (Tong *et al.* 2019)

No	Item	Descriptor and/or *examples*	Application
**A**	**Context and scope**		
1	Define geographical scope	Global, regional, national, city, local area, institutional/organisational level, health service	Organisational level of the Infection Prevention Society (IPS) members incorporating the United Kingdom, the Republic of Ireland and some international members.
2	Define health area, field, focus	Disease or condition specific, interventions, healthcare delivery, health system	Infection Prevention and Control (IPC) in healthcare
3	Define end-users of research	Intended beneficiaries e.g. general population or a specific population based on demographic (age, gender), clinical (disease, condition), or other characteristics	The aim is to provide guidance to the IPS and their members in relation to research priorities for the speciality and to inform the provision of research and quality improvement grant funding.
4	Define the target audience of the priorities	Policy makers, funders, researchers, industry	IPS members and IPC researchers
5	Identify the broad research area	Public health, health services research, clinical research, basic science	Infection prevention health services research
6	Identify the type of research question	Etiology, diagnosis, prevention, treatment (interventions), prognosis, health services, psychosocial, behavioral and social science, economic evaluation, implementation	Research focus
7	Define the time frame	Interim, short-term, long-term priorities, plans to revise and update	Long term priority is to utilise the findings to inform the research priorities and target financial support in those areas.
**B**	**Governance and team**		
8	Describe selection of the leadership and management team	Those responsible for initiating, developing, and guiding the process for priority setting e.g. Steering Committee, Advisory Group, Technical Experts	The leadership and management team are led by the Coordinator of the IPS Research and Development Committee
9	Describe the characteristics of the team, and the networks they represent	Stakeholder groups, organisations or networks represented, characteristics (demographics, experience, expertise)	The team are comprised of clinical and academic experts around IPC. They represent the research and development interests of the IPS and their members.
10	Describe any training or experience in priority setting	Consultants or advisors with experience in priority setting	The clinical members of the team are experienced on daily priority setting in clinical IPC including outbreak management and the academic members of the team are experienced in identification of research priorities and conduct of teaching and research.
**C**	**Framework for priority setting**		
11	State the framework used (if any)	James Lind Alliance, COHRED, CHNRI, no framework	Research and Evaluation Framework (Tan et al. 2022).

**D**	**Stakeholders or participants**		
12	Define the inclusion criteria for stakeholders involved in priority-setting	Patients, caregivers, general community, health professionals, researchers, policy makers, non-governmental organisations, government, industry; specific groups including vulnerable and marginalized populations	**Quantitative Research:** All participants were members of the IPS.<br>**Qualitative Research:** All participants were members of the Management Executive Group (MEG) and the Consultative Committee (CC) of the IPS.
13	State the strategy or method for identifying and engaging stakeholders	Partnership with organizations, social media, recruitment through hospitals	Engagement with IPS members via the MEG with the support of the IPS Secretariat
14	Indicate the number of participants and/or organisations involved	Number of individuals and organisations, include number by stakeholder group	IPS Membership: N = 2,415 (October 2022)<br>IPS MEG N = 16 and CC Membership: N = 24 (October 2022)
15	Describe the characteristics of stakeholders	Stakeholder group, demographic characteristics, areas of interest and expertise, discipline, affiliations	All stakeholders are working in or interested in IPC
16	State if reimbursement for participation was provided	Cash, vouchers, certificates, acknowledgement; what purpose e.g. travel, accommodation, honorarium	No reimbursement was provided to participants.
**E**	**Identification and collection of research priorities**		
17	Describe methods for collecting priorities from stakeholders	Methods e.g. Delphi survey, surveys, nominal group technique, interviews, focus groups, meetings, workshops; prioritization e.g. voting, ranking; mode e.g. face-to-face, online; may be informed by evidence e.g, systematic reviews, reviews of guidelines/other documents	Quantitative: Survey<br>Qualitative: Focus Group
18	Describe methods for collating and categorizing priorities	Taxonomy or other framework used to organise, summarise, and aggregate topics or questions	Mixed Methods Convergent Analysis, (Cresswell and Plano Clarke, 2011).
19	Describe methods and reasons for removing priorities	Based on scope, clarity, definition, duplication, other criteria	Based on exploration of topics during the focus group. Content that was nominal and not expanded on by the participants was not included in the qualitative results.
20	Describe methods for refining or translating priorities into research topics or questions	Reviewed by Steering Committee or project team	All results were reviewed by two researchers for the initial analysis (MS/EB) and then shared with two more researchers (AF/LK) for validation of the findings.
21	Describe methods for checking whether research questions or topics have been answered	Systematic reviews, evidence mapping, consultation with experts	The aim of this research was to identify topic areas as opposed to answering topic specific questions.
22	Describe number of research questions or topics	Number of priorities at each stage of the process	Eight research priorities were identified from the peer reviewed literature and were used to inform both the quantitative and qualitative research.

**F**	**Prioritisation of research topics/questions**		
23	Describe methods and criteria for prioritising research topics or questions	Methods e.g. Delphi survey, surveys, nominal group technique, interviews, focus groups, meetings, workshops; prioritisation e.g. voting, ranking; mode e.g. face-to-face, online; criteria e.g. need, feasibility, novelty, equity	Focus group
24	Provide reasons for excluding research topics/questions	Thresholds for ranking scores, proportions, votes; other criteria	Research themes that were not identified from the initial literature search were not included.
**G**	**Output**		
25	Specificity of research priorities are clear	Area, topic, questions, PICO (population, intervention, comparator, outcome)	The aim of this research was not to identify specific research questions but to identify specific research areas that are of value to the IPS to inform future research and funding.
**H**	**Evaluation and feedback**		
26	Describe how the process of prioritization was evaluated	Survey, workshop	Focus Group
27	Describe the approach for feeding back priorities to stakeholders and/or to the public; and how feedback was addressed and integrated	Public meetings or workshop, newsletters, website, email	The findings will be shared an oral presentation at the IPS conference 2023 and will be submitted for publication.
**I**	**Implementation**		
28	Outline the strategy or action plans for implementing priorities	Communication with target audience, via policies and funding	The findings will inform the provision of research grants from the IPS Research and Development Committee. All IPS members will be informed of same and the research supporting the decision.
29	Describe evaluation of impact	Integration in decision-making, funding allocation	Implementation must be approved by the IPS R&D Committee and the IPS MEG.
**J**	**Funding and conflict of interest**		
30	State sources of funding	Name sources of funding for the priority-setting exercise	IPS
31	Outline the budget and/or cost	Indicate budget and cost	Transcription / Statistics = £450.00
32	Provide declaration of conflict of interest	Statement of conflict of interest	There is no conflict of interest declared by the members of the research team.
