# Supplementary material for: Identifying research priorities for infection prevention and control. A mixed methods study with a convergent design
Source: J Infect Prev. 2024 Feb 20;25(3):59–65. doi: 10.1177/17571774241230676 (PMC10998549; doi:10.1177/17571774241230676)
Supplement: Supplemental Material - Identifying research priorities for infection prevention and control. A mixed methods study with a convergent design [file sj-pdf-4-bji-10.1177_17571774241230676.pdf]

Supplemental File 4: Consolidated criteria for reporting qualitative studies (COREQ), (Tong *et al.* 2007)

No. Item	Guide questions/description	Reported on Page #
**Domain 1: Research team and reflexivity**		
Personal Characteristics		
1. Inter viewer/facilitator	Which author/s conducted the interview or focus group?	MS / AF conducted the focus group.
2. Credentials	What were the researcher's credentials? E.g. PhD, MD	Supplemental File 1 (Reflexivity statement).
3. Occupation	What was their occupation at the time of the study?	Supplemental File 1 (Reflexivity statement).
4. Gender	Was the researcher male or female?	Supplemental File 1 (Reflexivity statement).
5. Experience and training	What experience or training did the researcher have?	Supplemental File 1 (Reflexivity statement).
Relationship with participants		
6. Relationship established	Was a relationship established prior to study commencement?	Some members of the quantitative research may have been known by the researchers and the qualitative focus group participants were known to the researchers. Page 2 Main Manuscript.
7. Participant knowledge of the interviewer	What did the participants know about the researcher? e.g. personal goals, reasons for doing the research	Participants were informed of the aim of the research via a participant information leaflet (quantitative) and a presentation (qualitative). Page 2 Main Manuscript.
8. Interviewer characteristics	What characteristics were reported about the inter viewer/facilitator? e.g. Bias, assumptions, reasons and interests in the research topic	Supplemental File 1 (Reflexivity statement).

**Domain 2: study design**		
Theoretical framework		
9. Methodological orientation and Theory	What methodological orientation was stated to underpin the study? e.g. grounded theory, discourse analysis, ethnography, phenomenology, content analysis	Mixed Methods (Cresswell and Plan Clarke) Page 2 Main Manuscript. Thematic Analysis (Braun and Clarke) Page 3 Main Manuscript.
Participant selection		
10. Sampling	How were participants selected? e.g. purposive, convenience, consecutive, snowball	Convenience Sampling via IPS meeting, Page 2 Main Manuscript.
11. Method of approach	How were participants approached? e.g. face-to-face, telephone, mail, email	Via IPS meeting, Page 2 Main Manuscript.
12. Sample size	How many participants were in the study?	21 members from the IPS Management Executive Group and Consultative Committee Group, Page 5 Main Manuscript.
13. Non-participation	How many people refused to participate or dropped out? Reasons?	No meeting attendee refused to participate.
Setting		
14. Setting of data collection	Where was the data collected? e.g. home, clinic, workplace	The qualitative focus group was held at a pre IPS conference meeting in Bournemouth, October 2022.
15. Presence of non-participants	Was anyone else present besides the participants and researchers?	Some members of the IPS Management Executive Group were present that did not participate as non-clinical.
16. Description of sample	What are the important characteristics of the sample? e.g. demographic data, date	Presented on Page 5, Main Manuscript – Table 4.
Data collection		

17. Interview guide	Were questions, prompts, guides provided by the authors? Was it pilot tested?	Question categories were focussed on the literature and were presented to the participants using a Power Point presentation before the focus group.
18. Repeat interviews	Were repeat inter views carried out? If yes, how many?	**N/A**
19. Audio/visual recording	Did the research use audio or visual recording to collect the data?	Audio recording of focus group was implemented with participants identifying themselves as a number during the process group to protect anonymity Page 3 Main Manuscript.
20. Field notes	Were field notes made during and/or after the interview or focus group?	Field notes were made by AF during and post the focus group.
21. Duration	What was the duration of the inter views or focus group?	The focus group took place over 60 minutes.
22. Data saturation	Was data saturation discussed?	Data saturation was not discussed as there was limited time to conduct the focus group.
23. Transcripts returned	Were transcripts returned to participants for comment and/or correction?	The focus group transcript was not returned to participants for checking.
**Domain 3: analysis and findings**		
Data analysis		
24. Number of data coders	How many data coders coded the data?	Page 3 Main Manuscript.
25. Description of the coding tree	Did authors provide a description of the coding tree?	N/A
26. Derivation of themes	Were themes identified in advance or derived from the data?	A framework of potential themes was identified from the

		literature however themes were derived from the data, Page 3.
27. Software	What software, if applicable, was used to manage the data?	NVivo 12 was used. Page 3 Main Manuscript.
28. Participant checking	Did participants provide feedback on the findings?	No
Reporting		
29. Quotations presented	Were participant quotations presented to illustrate the themes/findings? Was each quotation identified? e.g. participant number	Page 5 – 6 Main Manuscript and Supplemental File 5.
30. Data and findings consistent	Was there consistency between the data presented and the findings?	Page 5 – 9 Main Manuscript
31. Clarity of major themes	Were major themes clearly presented in the findings?	Page 5 – 9 Main Manuscript
32. Clarity of minor themes	Is there a description of diverse cases or discussion of minor themes?	Page 5 – 9 Main Manuscript
