# Supplementary material for: Identifying research priorities for infection prevention and control. A mixed methods study with a convergent design
Source: J Infect Prev. 2024 Feb 20;25(3):59–65. doi: 10.1177/17571774241230676 (PMC10998549; doi:10.1177/17571774241230676)
Supplement: Supplemental Material - Identifying research priorities for infection prevention and control. A mixed methods study with a convergent design [file sj-pdf-5-bji-10.1177_17571774241230676.pdf]

Supplemental File 5. Qualitative Themes, Findings and Supportive Quotations

Theme	Related Result	Quotation
**Patient Centred Care**	Patient centred care	*"Putting the patient at the heart of everything that we do",* (P1).
	Impact of the COVID-19 pandemic.	*"Because I think obviously when we had to react so much to Covid, I think a lot of things have been put on the backburner, but actually what has been the impact of not doing those things, but also what does that ideal service look like if we are putting the patient at the centre of our care?"* (P11).
**Training and Education**	**Healthcare Workers** Impact of the COVID-19 pandemic on T&E.	*"So, the problem is with Covid, I think a lot of the basics of IPC have been lost"* (P2).
	Informal T&E.	*"We should be saying to them, we'll support you, we'll help you, and if they know what's going on and we're doing it with them, we're going to get a lot more interaction from members and they're going to get a lot more support"* (P1).
	**IPC Practitioners** Professional Development and Retention.	*"How are we modernising as well to make IPC something worth looking at coming into",* (P12).<br><br>*"Advanced Nurse Practitioner… you have to register to actually undertake specific courses to be able to use that title, and how does IPC play into that? Because a lot of other courses are now incorporating that leadership module so that you would get the desired qualification to be able to use that title, but I'm not aware of any IPC course that's currently doing that",* (P2).
**IPC Role and Identity**	Impact of the COVID-19 pandemic on IPC Role and Identity.	*"What does that ideal service look like from an IPC point of view, and in terms of what the actual functions of that IPC team are given that we've had the pandemic, so what are the things you cannot lose if we ever had to have another pandemic?"* (P11).
	Diversity of IPC professionals.	*"We all see each other. Somebody might have said they're an Infection Control Nurse, somebody else said an Infection Prevention Nurse, so it's what are those priorities for individuals… how we see ourselves",* (P5).
	Autonomy and identity.	*"You can do this, you can't do that, you can only do this on this day, you can only do that on that day," and we have lost our way. And we're all very much senior practitioners in this room, and I think sometimes our clinical judgement, we haven't used it because we've been so swamped by this new "This is what you've got to do,""* (P1).

**IPC Leadership**	Multidisciplinary leadership role.	*"We can't work as a silo, we've got a lot of affiliations with a lot of other people in a lot of other societies, and isn't what we're about sharing our knowledge, sharing our expertise and making things better?",* (P1).
	Inclusion of IPC into integrated care systems.	*"I'm thinking it's that wider leadership but now bringing in the integrated care systems, so how do we get that same message across the wider organisations but allow each of the ICS's* (integrated care system) *to have their own priorities?* (P5).
**IPC is Everyone's Responsibility**	Lack of autonomy and accountability from non-IPC healthcare workers.	*"They [healthcare workers] love a guidance, they love a sheet that they can follow black and white. They hate going back to having to make decisions around clinical care and IPC. They also see very much as it's our business, not their business, so how we change that behaviour back to those practitioners and those clinicians making their own decisions about things, because we've actually had it the other way, that they want to still have rigid guidance, they don't want to take it back onto themselves",* (P13).
	Integration of IPC into routine patient care.	*"It's the fundamentals of the nursing care, that it's not IPC that's separate, this is about doing mouth care, washing a patient before they go to theatre, so it's how we bring it all back together",* (P9).
**IPC Research Activity**	Implementation of research.	*"So, it's actually having dedicated time to carry out the research and then implement the findings from it, to make it not just a paper exercise. Once you've done it and you've got that, it does encourage you to do it more and more",* (P2).
	Supportive organisational culture.	*"So, I've worked in a couple of organisations where there's been some very proactive research … but I know that that's not an opportunity that's available to a lot of people, so I think it really depends on where you've worked",* (P1).
	Time, confidence, and lack of support.	*"We've all got jobs, haven't we… I think that's what we need to overcome. How do we overcome that? Do we pay for somebody? Do we look for people with that have interest, who have time? It's a hard, hard thing to do",* (P5).
	Training of IPC in research	*"So, if that is part of our job descriptions, how do we understand the true meaning* [of research] *and true way it should be carried out?"* (P5).
